# Supplementary material for: Causal Relationship between Aging and Anorexia Nervosa: A White-Matter-Microstructure-Mediated Mendelian Randomization Analysis
Source: Biomedicines. 2024 Aug 16;12(8):1874. doi: 10.3390/biomedicines12081874 (PMC11351342; doi:10.3390/biomedicines12081874)
Supplement: Supplementary file 1 [file biomedicines-12-01874-s001.zip › Supplementary Figure/Figure S4/Singe SNP plot of MR analysis for TL to ubm-b-1799.pdf]

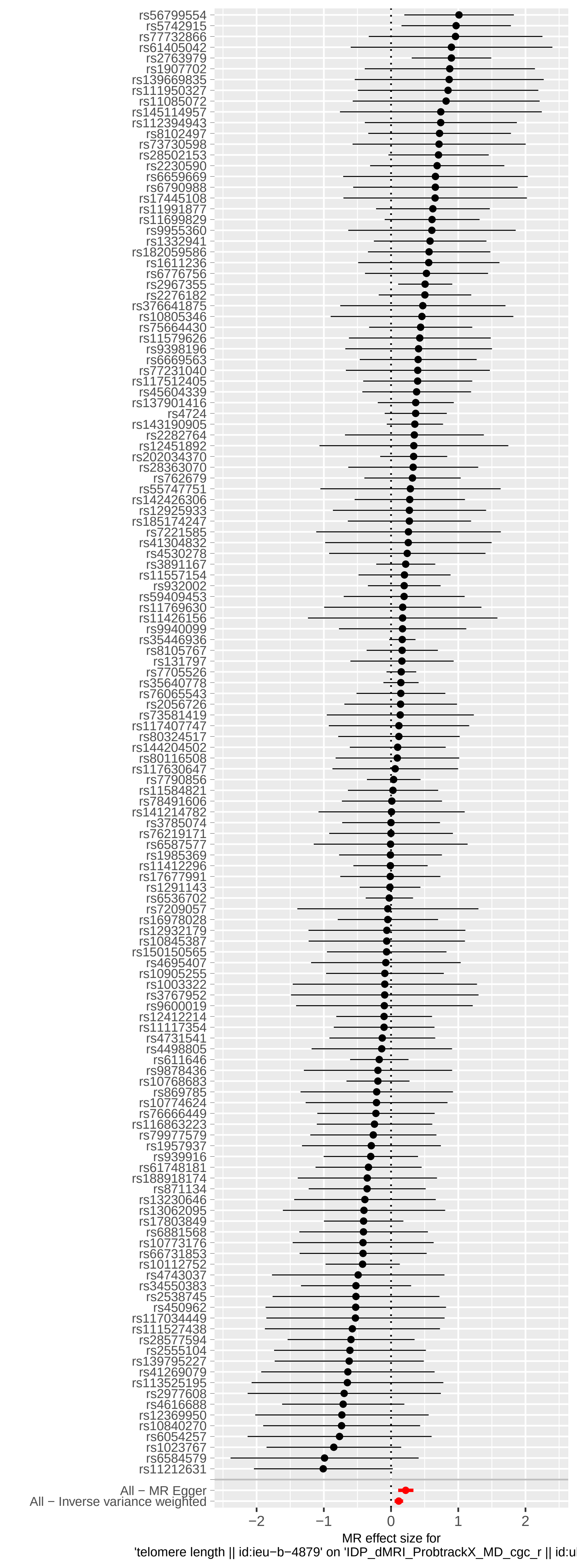
rs56799554
rs5742915
rs77732866
rs61405042
rs2763979
rs1907702
rs139669835
rs111950327
rs11085072
rs145114957
rs112394943
rs8102497
rs73730598
rs28502153
rs2230590
rs6659669
rs6790988
rs17445108
rs11991877
rs11699829
rs9955360
rs1332941
rs182059586
rs1611236
rs6776756
rs2967355
rs2276182
rs376641875
rs10805346
rs75664430
rs11579626
rs9398196
rs6669563
rs77231040
rs117512405
rs45604339
rs137901416
rs4724
rs143190905
rs2282764
rs12451892
rs202034370
rs28363070
rs762679
rs55747751
rs142426306
rs12925933
rs185174247
rs7221585
rs41304832
rs4530278
rs3891167
rs11557154
rs932002
rs59409453
rs11769630
rs11426156
rs9940099
rs35446936
rs8105767
rs131797
rs7705526
rs35640778
rs76065543
rs2056726
rs73581419
rs117407747
rs80324517
rs144204502
rs80116508
rs117630647
rs7790856
rs11584821
rs78491606
rs141214782
rs3785074
rs76219171
rs6587577
rs1985369
rs11412296
rs17677991
rs1291143
rs6536702
rs7209057
rs16978028
rs12932179
rs10845387
rs150150565
rs4695407
rs10905255
rs1003322
rs3767952
rs9600019
rs12412214
rs11117354
rs4731541
rs4498805
rs611646
rs9878436
rs10768683
rs869785
rs10774624
rs76666449
rs116863223
rs79977579
rs1957937
rs939916
rs61748181
rs188918174
rs871134
rs13230646
rs13062095
rs17803849
rs6881568
rs10773176
rs66731853
rs10112752
rs4743037
rs34550383
rs2538745
rs450962
rs117034449
rs111527438
rs28577594
rs2555104
rs139795227
rs41269079
rs113525195
rs2977608
rs4616688
rs12369950
rs10840270
rs6054257
rs1023767
rs6584579
rs11212631
All − MR Egger
All − Inverse variance weighted
−2
−1
0
1
2
MR effect size for
'telomere length || id:ieu−b−4879' on 'IDP_dMRI_ProbtrackX_MD_cgc_r || id:u
